# Supplementary material for: Membrane remodeling properties of the Parkinson’s disease protein LRRK2
Source: Proc Natl Acad Sci U S A. 2023 Oct 16;120(43):e2309698120. doi: 10.1073/pnas.2309698120 (PMC10614619; doi:10.1073/pnas.2309698120)
Supplement: Supplementary file 1 — Appendix 01 (PDF) [file pnas.2309698120.sapp.pdf]

Supporting Information

Membrane remodeling properties of the Parkinson's disease protein LRRK2

Xinbo Wang[1,2,3,4,6], Javier Espadas[7], Yumei Wu[1,2,3,4,6], Shujun Cai[1,2,3,4,6], Jinghua Ge[2], Lin Shao[1,5], Aurélien Roux[7], Pietro De Camilli[1,2,3,4,5,6*]

[1]Department of Neuroscience, Yale University School of Medicine, New Haven, Connecticut 06510, USA;

[2]Department of Cell Biology, Yale University School of Medicine, New Haven, Connecticut 06510, USA;

[3]Howard Hughes Medical Institute, Yale University School of Medicine, New Haven, Connecticut 06510, USA;

[4]Program in Cellular Neuroscience, Neurodegeneration and Repair, Yale University School of Medicine, New Haven, Connecticut 06510, USA;

[5]Kavli Institute for Neuroscience, Yale University School of Medicine, New Haven, Connecticut 06510, USA;

[6]Aligning Science Across Parkinson's (ASAP) Collaborative Research Network, Chevy Chase, MD, 20815, USA.

[7]Department of Biochemistry, University of Geneva, Geneva, CH-1211, Switzerland.

[*]Correspondence to: pietro.decamilli@yale.edu

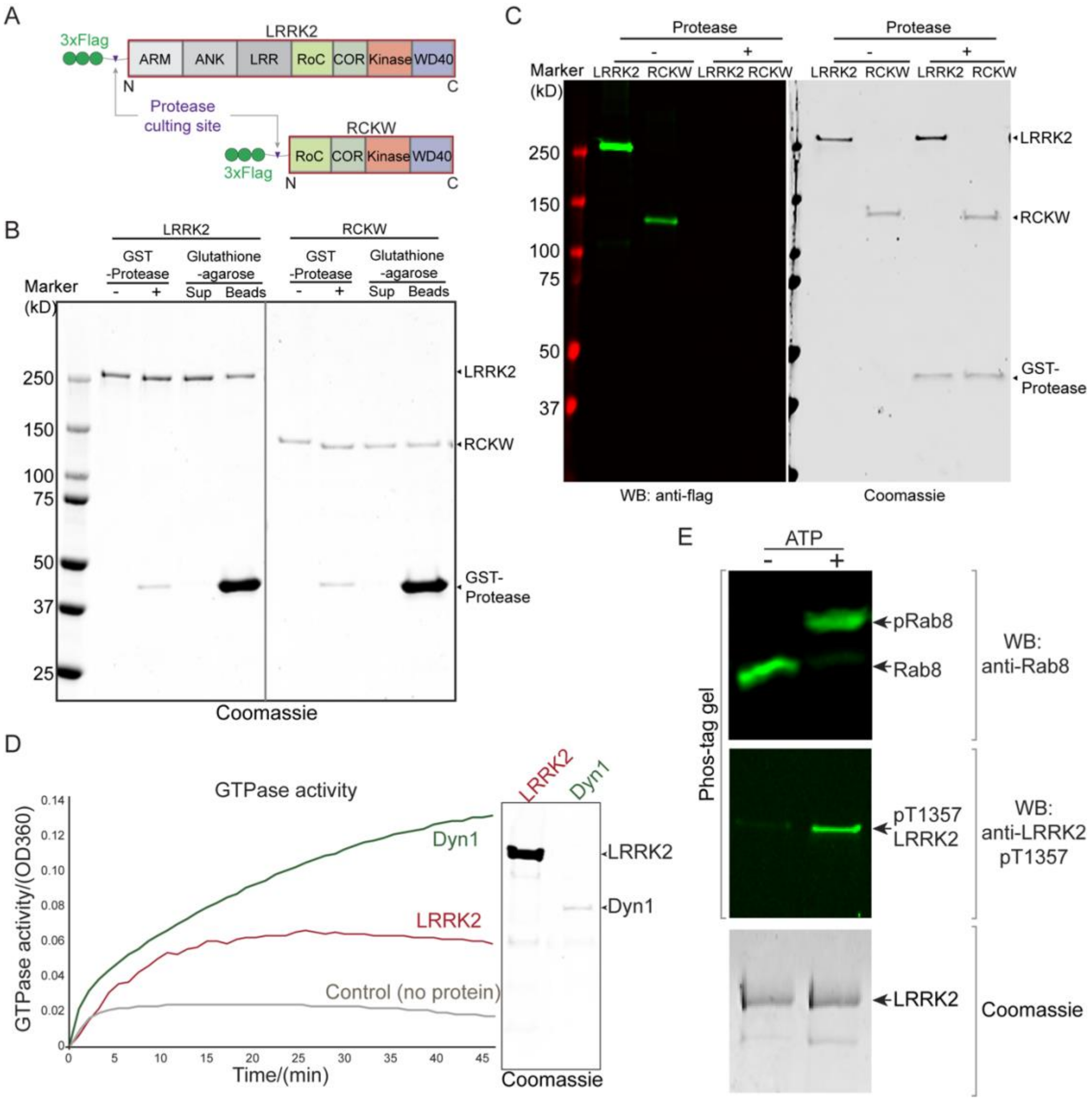

Fig. S1. Purified LRRK2 is enzymatically active. (A) Domain cartoon of the purified proteins used in the functional assays. (B) Coomassie-stained SDS gels showing fractions obtained during the purification of recombinant full-length LRRK2 and RCKW fragment from Expi293 cells. Proteins were purified on anti-flag M2 resin, eluted from the beads using the FLAG peptide, followed by GST-Protease to remove the tag and further purified by removing the GST-protease by Glutathione-agarose. LRRK2- and RCKW-containing supernatants (Sup) were dialyzed before use. (C) Anti-FLAG tag Western blot (WB) and Coomassie-stained gel showing successful removal of the flag tag. (D) *In vitro* GTPase activity assay. The GTPase activity of LRRK2 (9 µM) and dynamin 1 (Dyn 1) (0.8 µM) were measured by phosphate release at saturating concentrations of GTP (0.5 mM). Left, LRRK2 has detectable GTPase activity but a much lower activity than dynamin 1. Right, Coomassie-stained SDS-PAGE showing the proteins used at the same molar ratio used for the GTPase assay. (E) Protein kinase activity assay. LRRK2-mediated phosphorylation of recombinant Rab8 (upper), and autophosphorylation of LRRK2 (middle), were analyzed by Phos-tag gels using an anti-Rab8 or an anti-LRRK2 phospho-specific (pT1357) antibody, respectively. Coomassie Blue stained gels of LRRK2 samples used for the assay are shown at the bottom of the figure.

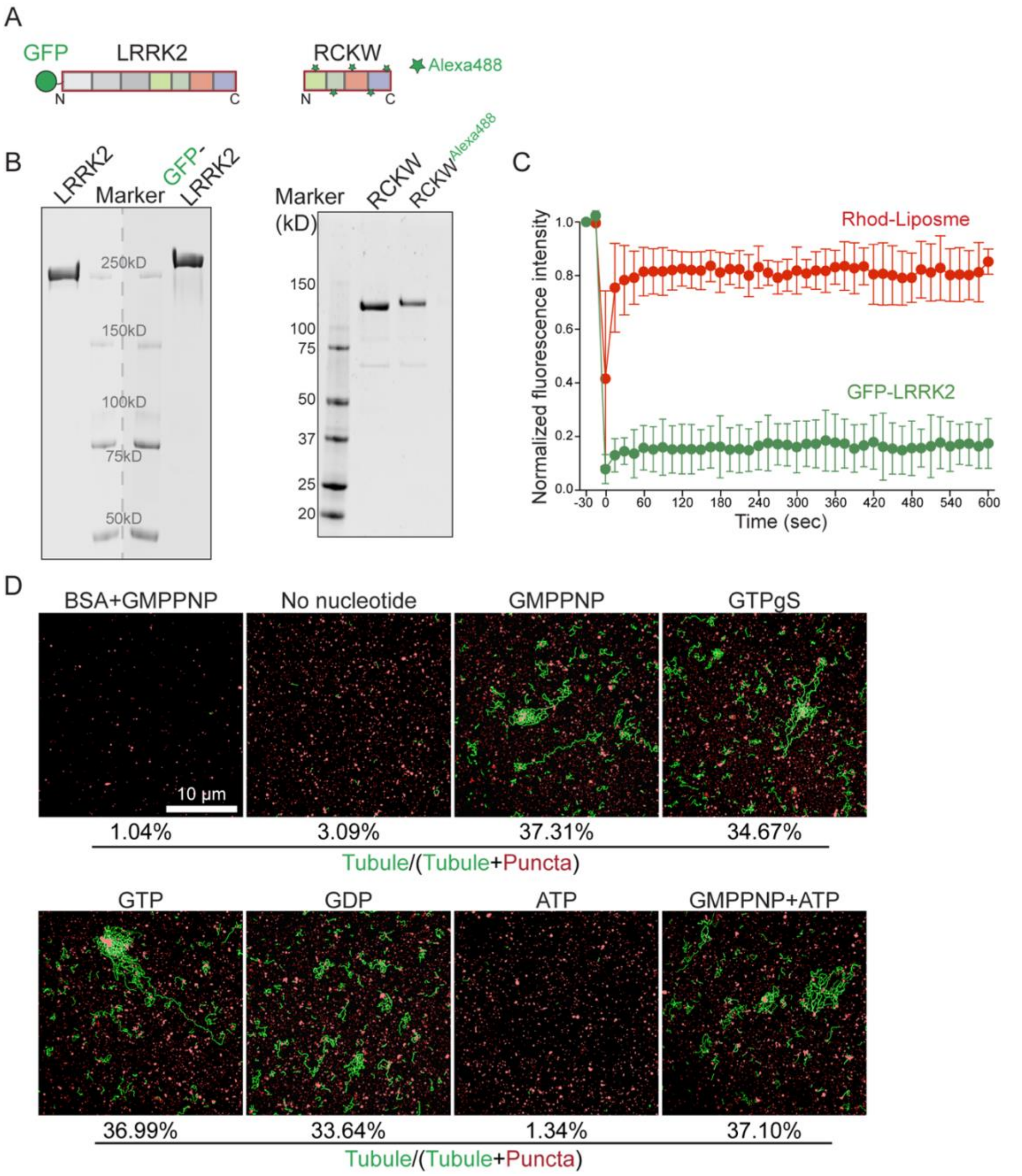

Fig. S2. Characterization of LRRK2-induced liposome tubulation. (A) Proteins used in the tubulation assays. (B) Coomassie Blue-stained SDS-gels showing tagged proteins used for the assays and, as a control of motility, the corresponding untagged proteins. (C) Plot of the average fluorescence intensities after photobleaching of multiple ~2mm segments on different tubules. Normalized fluorescence intensities are plotted versus time (s). Values represent mean ± S.D (n=3). (D) Representative processed images highlighting LRRK2-induced liposome tubulation in the absence or presence of different nucleotides. Values below the images represent the percent area of total liposome fluorescence accounted for by the tubules in the micrograph shown.

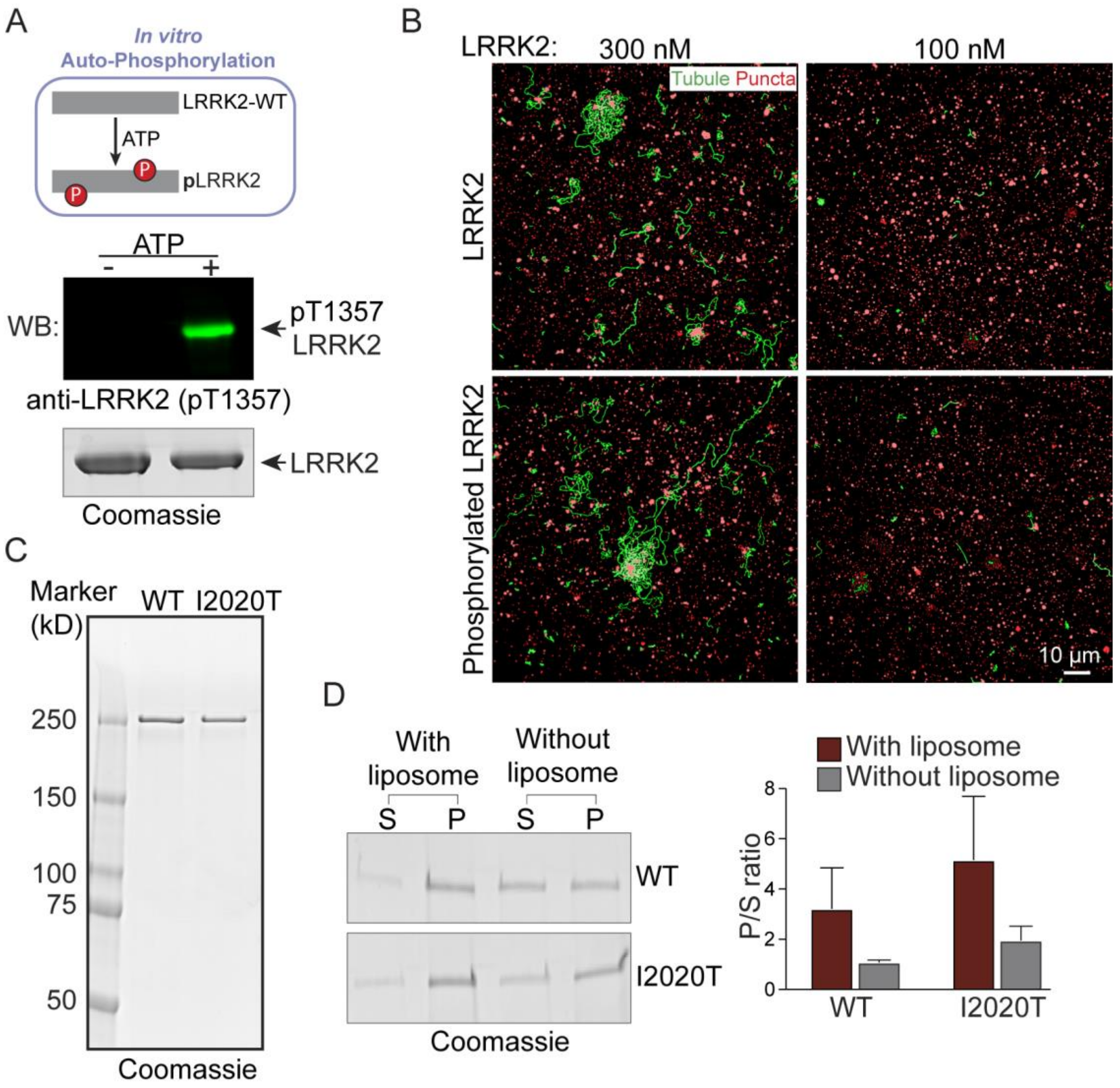

Fig. S3. ***In vitro* LRRK2 autophosphorylation and liposome binding of mutant LRRK2.** (A) *In vitro* LRRK2 autophosphorylation assay. LRRK2 autophosphorylation was initiated by adding 1mM ATP to the phosphorylation mixture and validated by Western blotting using a LRRK2 phospho-specific (pT1357) antibody. A Coomassie blue stained gel of the LRRK2 proteins used is shown at the bottom. (B) Processed images of liposome tubules induced by LRRK2 (top panels) or autophosphorylated LRRK2 (bottom panels). (C) Coomassie blue-stained gel showing purified wild type (WT) and mutant LRRK2 (I2020T) proteins. (D) Left, Coomassie blue-stained gel of pellets (P) and supernatants (S) of liposomes incubated in the presence or absence of full length wild-type (WT) or mutant (I2020T) LRRK2 and then subjected to centrifugation. Right, Quantification of the intensities of gel bands in pellets and supernatants (n=3). Bars represents ratios between proteins present in the pellets and in the supernatant. Values represent mean ± S.D (n=3).

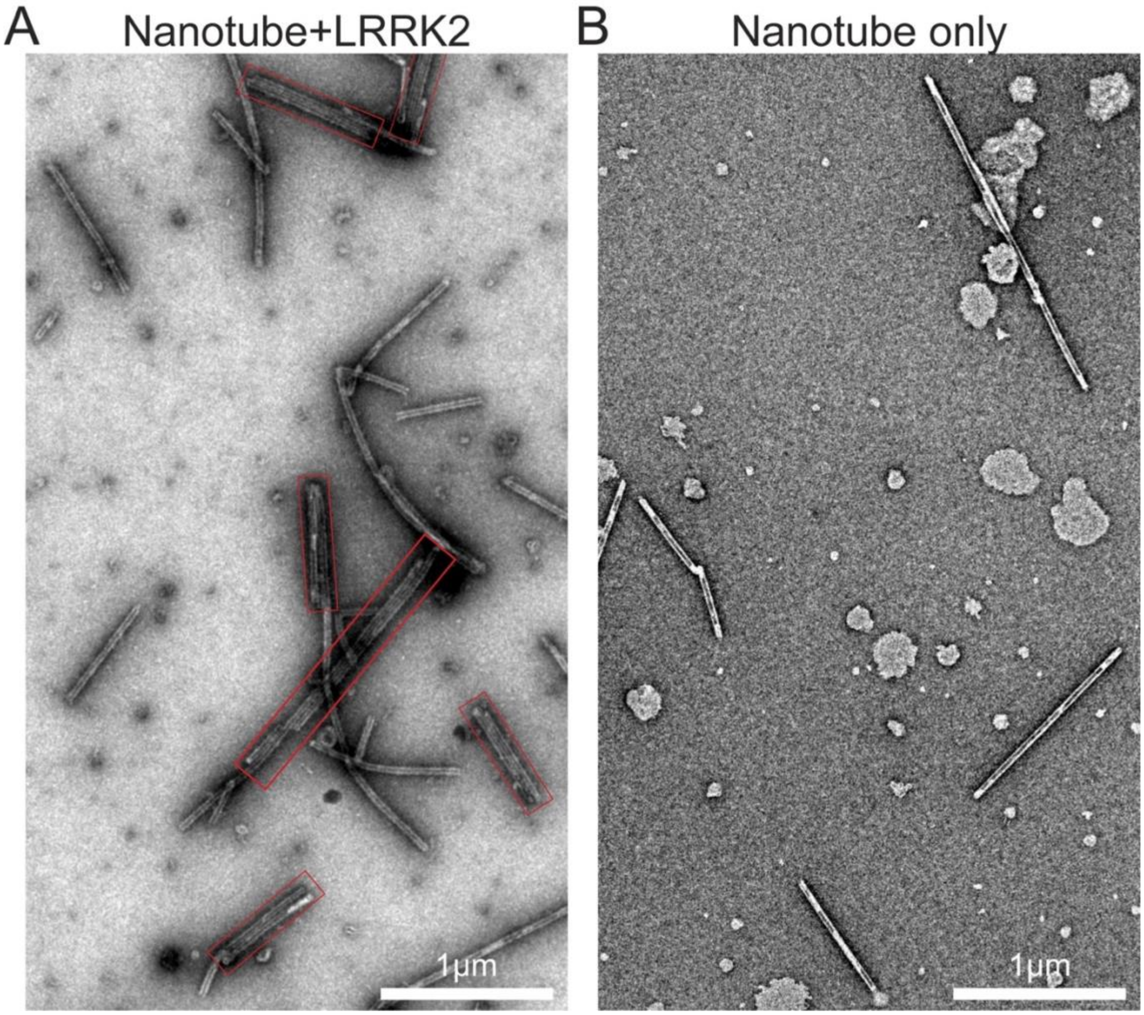

Fig. S4. LRRK2 binds to lipid nanotubes. (A) Low magnification EM images of negatively stained nanotubes incubated in the presence of LRRK2. Note presence of nanotube bundles induced by LRRK2 (red boxes). (B) Nanotube only control.

Movie S1 (separate file). Confocal time-lapse imaging showing Rhod-labeled liposome tubulation induced by GFP-LRRK2. Images were collected at a rate of 1 frame every 20 seconds. Scale bars, 10 µm.
